# Ferritin‐based disruptor nanoparticles: A novel strategy to enhance LDL cholesterol clearance via multivalent inhibition of PCSK9–LDL receptor interaction

**DOI:** 10.1002/pro.5111

**Published:** 2024-08-16

**Authors:** Alessio Incocciati, Chiara Cappelletti, Silvia Masciarelli, Francesca Liccardo, Roberta Piacentini, Alessandra Giorgi, Lucia Bertuccini, Barbara De Berardis, Francesco Fazi, Alberto Boffi, Alessandra Bonamore, Alberto Macone

**Affiliations:** ^1^ Department of Biochemical Sciences “Alessandro Rossi Fanelli” Sapienza University of Rome Rome Italy; ^2^ Department of Anatomical, Histological, Forensic & Orthopaedic Sciences, Section of Histology and Medical Embryology, Laboratory Affiliated to Istituto Pasteur Italia‐Fondazione Cenci Bolognetti Sapienza University of Rome Rome Italy; ^3^ Center for Life Nano Science at Sapienza Istituto Italiano di Tecnologia Rome Italy; ^4^ Core Facilities, Microscopy Area, Istituto Superiore di Sanita Rome Italy; ^5^ National Center for Innovative Technologies in Public Health Istituto Superiore di Sanità Rome Italy

**Keywords:** alirocumab, evolocumab, ferritin, HepG2 cell line, hypercholesterolemia, LDL cholesterol, LDL receptor, PCSK9, protein nanoparticles, therapeutic peptides

## Abstract

Hypercholesterolemia, characterized by elevated low‐density lipoprotein (LDL) cholesterol levels, is a significant risk factor for cardiovascular disease. Proprotein convertase subtilisin/kexin type 9 (PCSK9) plays a crucial role in cholesterol metabolism by regulating LDL receptor degradation, making it a therapeutic target for mitigating hypercholesterolemia‐associated risks. In this context, we aimed to engineer human H ferritin as a scaffold to present 24 copies of a PCSK9‐targeting domain. The rationale behind this protein nanoparticle design was to disrupt the PCSK9–LDL receptor interaction, thereby attenuating the PCSK9‐mediated impairment of LDL cholesterol clearance. The *N*‐terminal sequence of human H ferritin was engineered to incorporate a 13‐amino acid linear peptide (Pep2‐8), which was previously identified as the smallest PCSK9 inhibitor. Exploiting the quaternary structure of ferritin, engineered nanoparticles were designed to display 24 copies of the targeting peptide on their surface, enabling a multivalent binding effect. Extensive biochemical characterization confirmed precise control over nanoparticle size and morphology, alongside robust PCSK9‐binding affinity (*K*_D_ in the high picomolar range). Subsequent efficacy assessments employing the HepG2 liver cell line demonstrated the ability of engineered ferritin's ability to disrupt PCSK9–LDL receptor interaction, thereby promoting LDL receptor recycling on cell surfaces and consequently enhancing LDL uptake. Our findings highlight the potential of ferritin‐based platforms as versatile tools for targeting PCSK9 in the management of hypercholesterolemia. This study not only contributes to the advancement of ferritin‐based therapeutics but also offers valuable insights into novel strategies for treating cardiovascular diseases.

## 1 INTRODUCTION

Ferritin, a widely distributed iron‐storage protein, plays a pivotal role in the regulation of iron homeostasis by forming a protective shell composed of 24 units that sequester this metal ion preventing its toxic accumulation. This unique structure, combined with its biocompatibility, biodegradability, and low immunogenicity, makes it an attractive platform for developing therapeutic nanoparticles (Lee et al., 2022; Song et al., 2021). Indeed, its hollow, spherical shape can be used in its native form or customized through chemical or genetic modification to encapsulate a wide range of therapeutic agents, including drugs (Huang et al., 2021; Incocciati et al., 2023), proteins (Macone et al., 2019; Tetter & Hilvert, 2017), nucleic acids (Li et al., 2016; Palombarini et al., 2021), and imaging agents (Calisti et al., 2018; Sitia et al., 2020). Ferritin can also be modified on its external surface to selectively target specific cells or tissues, thereby improving drug delivery efficiency and reducing off‐target effects (Ma, Dong, et al., 2021; Ma, Li, et al., 2021; Sevieri et al., 2023). This can be achieved through *N*‐terminal modifications, as they do not significantly affect the assembly of the nanoparticle, within certain limits. The *N*‐terminus sequence can also be engineered to present antigens, eliciting an immune response and offering potential for the development of ferritin‐based vaccines against various diseases (Houser et al., 2022; Joyce et al., 2022; Vu et al., 2023). Hence, ferritin can be modified to specifically recognize a wide range of molecular targets with strong affinity, and as it displays multiple copies (up to 24) of a specific peptide sequence on its surface, the likelihood of target recognition and binding can be very high.

In this study, we developed a variant of human H ferritin (HFn) that contains multiple copies of a domain, capable of binding to proprotein convertase subtilisin/kexin type 9 (PCSK9) on its surface. PCSK9 is a critical protein involved in the posttranslational regulation of cholesterol metabolism and is primarily synthesized in the liver (Seidah & Prat, 2022). It functions to modulate circulating levels of low‐density lipoprotein cholesterol (LDL‐C). Elevated levels of PCSK9, or “gain of function” variants, have been associated with an increased risk of cardiovascular disease and familial hypercholesterolemia (Libby & Tokgözoğlu, 2022; Shapiro et al., 2018), while “loss of function” mutations are associated with low LDL‐C levels, a decreased risk of cardiovascular events and, notably, no associated adverse consequences (Cohen et al., 2006; Zhao et al., 2006). Consequently, PCSK9 has emerged as a key therapeutic target for lowering circulating LDL‐C and preventing atherosclerotic cardiovascular diseases.

The mechanism of action of PCSK9 in hypercholesterolemia involves its binding to the LDL receptor (LDLR) on the surface of liver cells (Kwon et al., 2008). This interaction occurs at a specific domain of LDLR called the EGF‐A (epidermal growth factor precursor homology domain A). Under normal conditions, LDLR removes LDL‐C from the bloodstream by binding to it and facilitating its uptake into the liver for degradation and excretion. Upon internalization, both LDL‐C and LDLR are separated within the cell: LDL‐C is transported to the lysosome for degradation, while LDLR is recycled back to the cell surface to continue its role in removing LDL‐C from the bloodstream. The LDLR undergoes recycling approximately every 10 min and has a lifespan of approximately 20 h, allowing it to effectively internalize multiple LDL particles during its lifetime. However, when PCSK9 binds to LDLR, it promotes its degradation into lysosomes and reduces the ability of LDLR to be recycled on the cell surface. This disrupts the normal process of LDL‐C clearance, leading to LDL‐C accumulation in the bloodstream, which is a significant risk factor for the development of cardiovascular diseases (Shapiro et al., 2018). Experimental and clinical research indicates that hypercholesterolemia is influenced not only by PCSK9 “gain‐of‐function” variants but also by the overexpression of the wild‐type protein. This overexpression, which can be correlated with multiple metabolic variables and systemic inflammation, may accelerate the development of atherosclerotic plaques (Bao et al., 2024).

Our engineered HFn variant aims to interfere with the interaction between PCSK9 and LDLR by presenting multiple copies of a PCSK9‐binding domain on the surface of ferritin, with the goal of mitigating the detrimental effects of PCSK9 on LDL‐C clearance and potentially offering a novel approach for the treatment of hypercholesterolemia (Jiang et al., 2018). Our approach involves displaying 24 copies of a 13‐amino acid linear peptide (Pep2‐8) on the surface of ferritin, with the objective of optimizing its solubility and effectiveness, leveraging the multivalence effect of ferritin. Accordingly, we addressed the genetic engineering of the *N*‐terminal sequence of the HFn subunits to incorporate Pep2‐8, which has been previously identified as the smallest peptide able to inhibit PCSK9 (Zhang et al., 2014). Given the promising potential of Pep2‐8 in the development of small peptide‐based PCSK9 inhibitors (Tombling et al., 2021), we aimed to address its limitations in terms of solubility and efficacy by genetically fusing it to human ferritin, a protein that naturally targets the liver, an organ that plays a crucial role in maintaining whole‐body cholesterol homeostasis. The resulting HFn‐Pep2‐8 nanoparticles were characterized using size exclusion chromatography, dynamic light scattering, and electron microscopy to determine their size and morphology. The binding properties of the nanoparticles to PCSK9 were evaluated using biolayer interferometry in order to assess the kinetics and affinity of the interaction. In addition to biochemical characterization, we assessed the efficacy of the engineered ferritin by examining its ability to bind and inhibit PCSK9 activity in HepG2 cells.

## 2 MATERIALS AND METHODS

### 2.1 Protein expression and purification

A sequence encoding the Pep2‐8 peptide followed by a four‐glycine flexible linker (TVFTSWEEYLDWVGGGG) was added to the 5′‐terminus of a synthetic gene encoding for human H ferritin (HFn). Both the HFn and HFn‐Pep2‐8 genes were optimized for the expression in *Escherichia coli* cells and subcloned into the pET22b vector. Protein expression was induced with 1 mM IPTG (isopropyl‐β‐d‐1‐thiogalactopyranoside) at an OD_600_ = 0.6 for 16 h at 37°C and 22°C for HFn and HFn‐Pep2‐8, respectively, and the cells were harvested by centrifugation.

#### 2.1.1 HFn purification protocol

Bacterial paste from a 1 L culture of HFn was resuspended in 50 mL of 20 mM sodium phosphate buffer, pH 7.4, containing 150 mM NaCl and protease inhibitors (Roche©) disrupted by sonication. The soluble fraction was treated with 50% and 70% (NH_4_)_2_SO_4_. The 70% (NH_4_)_2_SO_4_ pellet was resuspended in 20 mM sodium phosphate buffer, pH 7.4, containing 150 mM NaCl and extensively dialyzed against the same buffer overnight at 4°C. After dialysis, the protein sample was subjected to heat treatment at 75°C for 10 min. The resulting soluble fraction was digested with 50 μg/mL deoxyribonuclease I (Merck) for 1 h at 37°C with the addition of 2 mM MgCl_2_. After digestion, the protein sample was loaded onto a HiLoad 26/600 Superdex 200 pg column previously equilibrated with 20 mM sodium phosphate buffer (pH 7.4) containing 150 mM NaCl using an AKTA‐Pure apparatus (Cytiva). The protein fractions eluted at the retention time of ferritin were pooled, concentrated using Amicon Ultra15 centrifugal filter devices (100 kDa cutoff), sterile filtered, and stored at 4°C (50 mg of purified protein per liter of culture medium). The protein concentration was calculated by measuring the UV absorption at 280 nm (*ε*_280_ = 19,000 M^−1^ cm^−1^, extimated by ProtParam, Expasy), and the protein purity was checked by SDS‐PAGE and high‐performance size exclusion chromatography (HP‐SEC).

#### 2.1.2 HFn‐Pep2‐8 purification protocol

Bacterial paste from a 1 L culture of HFn‐Pep2‐8 was resuspended in 100 mL of 20 mM sodium phosphate buffer, pH 7.4, containing 150 mM NaCl, 0.5 mM TCEP, and protease inhibitors (Roche©). After sonication, HFn‐Pep2‐8 was recovered from the insoluble fraction and subjected to further sonication treatment (Neerathilingam et al., 2014): inclusion bodies were resuspended in 100 mL of 20 mM Tris buffer, pH 8.5, containing 0.5 mM TCEP and 1 M urea, and sonicated for 10 cycles (30 s pulse and 30 s pause) at 40% amplitude. The soluble fraction was recovered and diluted fourfold in 20 mM phosphate buffer containing 150 mM NaCl without urea to induce protein precipitation. The precipitate was resuspended in 8 mL of 20 mM sodium phosphate buffer (pH 7.4) containing 50 mM NaCl and loaded onto a HiLoad 26/600 Superdex 200 pg column equilibrated with the same buffer using an AKTA‐Pure apparatus (Cytiva). The protein fractions eluted at the retention time of ferritin were pooled, concentrated using Amicon Ultra15 centrifugal filter devices (100 kDa cutoff), sterile filtered, and stored at 4°C. The protein concentration was calculated by measuring the UV absorption at 280 nm (*ε*_280_ = 31,400 M^−1^ cm^−1^, estimated by ProtParam, Expasy), and the protein purity was checked by SDS‐PAGE and HP‐SEC.

### 2.2 High‐performance size exclusion chromatography

HFn and HFn‐Pep2‐8 purity, aggregation state, and stability were analyzed by high‐performance size exclusion chromatography (HP‐SEC). HP‐SEC analyses were performed by means of an Agilent Infinity 1260 HPLC apparatus equipped with UV detectors using an Agilent AdvanceBio SEC 300 Å 2.7 μm 4.6 × 150 mm column. Isocratic analysis was carried out with 20 mM sodium phosphate buffer (pH 7.4) containing 50 mM NaCl_2_ as the mobile phase. The flow rate was 0.7 mL/min over an elution window of 10 min. Ferritin elution was followed by UV detection at 220 and 280 nm. HFn‐Pep2‐8 stability was analyzed by HP‐SEC by recording the area of the protein peak for 8 h at 37°C in MEM.

### 2.3 Native polyacrylamide gel electrophoresis (PAGE)

Native polyacrylamide gel electrophoresis was performed with a 4%–15% nondenaturing acrylamide gel (Mini‐PROTEAN TGX stain‐free) in Tris‐glycine pH 8.3 running buffer at room temperature for 30–40 min at 150–200 V in a Bio‐Rad Mini‐Protean tetra‐cell electrophoresis apparatus.

### 2.4 MALDI TOF/TOF analysis

The interesting bands, corresponding to HFn and HFn‐Pep2‐8, were excised from SDS‐PAGE and subjected to tryptic proteolysis. After two steps for destaining and dehydrating with aqueous solutions of 50 mM ammonium bicarbonate with or without acetonitrile, the bands were reduced and alkylated with iodoacetamide. Proteolysis was carried out with Trypsin Gold (Mass Spectrometry Grade, Promega) at 37°C overnight. The tryptic mixtures were analyzed by an ultrafleXtreme MALDI ToFToF (Bruker, Bremen, Germany) instrument equipped with a Smartbeam‐II laser in positive and reflector modes. Tandem mass analyses in LIFT mode were performed to confirm protein identities.

### 2.5 Transmission electron microscopy (TEM) analysis

Ferritin solutions (15 μg/mL) were diluted 1:10 in buffer and analyzed by the drop‐on‐grid method: 5 μL of each sample was deposited on Formvar carbon‐coated grids, blotted gently with filter paper after 5 min, stained with 4% ammonium molybdate for 30 s, blotted again with filter paper, and air‐dried. The grids were analyzed at 100 kV by an EM208S transmission electron microscope (FEI—Thermo Fisher Scientific; Eindhoven—The Netherlands) equipped with a Megaview II SIS camera (Olympus‐SIS Milan, Italy). The free software ImageJ (version 1.29; NIH, Bethesda, MD) was used to analyze high‐magnification micrographs for calculating the diameter size distribution of the ferritin globulins. In particular, manual measurements of more than 100 particles were conducted for each sample and the diameter size distributions were calculated through Excel 2016 software.

HFn‐Pep2‐8/PCSK9 complex was analyzed as described above after incubating HFn‐Pep2‐8 with 10 molar excess of PCSK9 (Merck, code: SRP6285) for 2 h at room temperature.

### 2.6 Dynamic light scattering (DLS) characterization and zeta potential

Protein suspensions in PBS at 0.1 mg/mL were characterized by a Zetasizer Ultra instrument (Malvern Instrument, UK) equipped with two technologies, noninvasive back scattering (NIBS) and multi‐angle dynamic light scattering (MADLS), to determine the hydrodynamic diameter, size distribution, and particle number concentration. DLS measurements were performed on 1 mL of the suspensions. The equilibration step at 25°C was set at 2 min. Three determinations were performed on each sample. The instrument software automatically determined the number read and duration of each determination. To determine the hydrodynamic diameter (*Z*‐average) and polydispersity index, data related to distributions by intensity were analyzed. The intensity‐weighted size distribution was determined by ZS Xplorer Software (Malvern Instruments, UK).

For the evaluation of the particle number concentration, the particle size distribution obtained by MADLS was determined by ZS Xplorer software to calculate the scattering cross‐section and the amount of scattering per particle and then to convert the total scattering detected from the sample into the number of particles per mL. The surface charge of 0.1 mg/mL protein suspensions in PBS was assessed via electrokinetic measurements by Zetasizer Ultra Instrument (Malvern Instrument, UK) incorporating an electrophoretic light scattering system. The electrophoretic mobility values, obtained by the 3 M‐PALS technique in constant current mode, were converted to zeta potentials using an automatic instrument measurement protocol. The measurements were conducted in triplicate on 750 μL of nanoparticle suspensions.

### 2.7 Biolayer interferometry

Biolayer interferometry (BLI) assays were employed to evaluate the affinity of HFn‐Pep2‐8 for both PCSK9 and the transferrin receptor 1 (CD71) using the Octet N1 system (Sartorius). His‐tagged PCSK9 or His‐tagged CD71 was immobilized on a biosensor tip (Ni‐NTA), and various concentrations of HFn‐Pep2‐8 were assessed within the ranges of 25–800 nM for PCSK9 and 2.5–1580 nM for CD71. Before each analysis, biosensors underwent a 10‐min equilibration period in 1× kinetic buffer (PBS containing 0.02% Tween 20, 0.1% BSA, and 0.05% NaN_3_). Following equilibration, the biosensor tip was placed in the HFn‐Pep2‐8 solution, and the duration of each measurement was optimized to ensure maximal binding capacity. The recorded data were subsequently analyzed using Octet software to determine the kinetic parameters. All association/dissociation curves were fitted using single exponential growth and decay functions. The plateau value derived from the single exponential growth function was utilized to calculate *K*_D_ values by employing the following equation: response = (*R*_max_*C)/*K* + *C*, where *R*_max_ = maximum response, *C* = concentration of HFn‐Pep2‐8, and *K* = dissociation constant. The reported *K*_D_ values represent the average and standard deviation of three independent experiments.

### 2.8 In vitro PCSK9/LDL receptor binding assay

The ability of HFn‐Pep2‐8 to interfere with the PCSK9–LDL receptor interaction was assessed using a CircuLex PCSK9‐LDLR in vitro binding assay kit (MBL, Japan) following the manufacturer's instructions. His‐tagged PCSK9 (at a final concentration of 70 ng/mL) or His‐tagged PCSK9 preincubated with HFn‐Pep2‐8 (at concentrations ranging from 1 to 280 nM) was added to each well of the microplate containing the immobilized LDLR‐AB domain. After a 2‐h incubation period, the wells were thoroughly washed, and biotinylated anti‐His‐tag monoclonal antibody was added and incubated for 1 h under shaking. Subsequently, HRP‐conjugated streptavidin was added, and the plate was further incubated for 20 min. After the final wash, the chromogenic substrate tetra‐methylbenzidine was added, and the reaction was halted with 2.0 N sulfuric acid. The absorbance at 450 nm was then measured using an Appliskan® multimode microplate reader (Thermo Scientific). The percent inhibition of the test compounds was calculated relative to that of the vehicle control (PCSK9 only), which was considered 100% binding. GraphPad Prism 10.0 software was used to fit the obtained measurements. The reported IC_50_ value represents the average and standard deviation of three independent experiments.

### 2.9 Cell culture and cell viability assay

The human hepatocellular carcinoma cell line HepG2 (NB‐19‐0060) was obtained from Neo Biotech (Seoul, Seoul‐t'ukpyolsi, South Korea) and cultured in MEM with Earle's Salts with Stable Glutamine (MEM‐STA) medium from Capricorn Scientific (Ebsdorfergrund, Germany) supplemented with 1% penicillin/streptomycin and 10% heat‐inactivated FBS (Gibco, Thermo Fisher Scientific, Waltham, MA, USA). The culture was maintained at 37°C in a humidified atmosphere containing 5% CO_2_. For the experiments, the cells were cultured in 24‐well plates (Corning Incorporated, NY, USA). When the cells reached 70% confluence, the growth medium was replaced with MEM supplemented with lipoprotein‐deficient serum from the fetal calves from Sigma‐Aldrich (St. Louis, MO, USA). After 24 h, the cells were treated with 0.1 μM or 1 μM HFn‐Pep2‐8 or with the same amount of the buffer in which HFn‐Pep2‐8 was solubilized. After 8 h, cell viability was tested by performing a propidium iodide exclusion assay (Sigma‐Aldrich, St. Louis, MO, USA). The percentages of cell death obtained by flow cytometry (Cytoflex Beckman Coulter, Life Sciences, Brea, CA, USA) were analyzed by CytExpert v2.2 software (Beckman Coulter).

### 2.10 LDL‐R membrane levels

HepG2 cells at 70% confluency, seeded on IBIDI μ‐Slide 8 Well high ibiTreat slides (IBIDI, Gräfelfing, Germany), were incubated for 24 h before starting the experiment with MEM supplemented with lipoprotein‐deficient serum from fetal calf and then treated with 7 μg/mL PCSK9 alone or in combination with HFn‐Pep2‐8 0.1 μM and HFn‐Pep2‐8 1 μM in serum‐free medium. After 8 h, the treated cells were washed with DPBS, fixed with 4% paraformaldehyde for 10 min (Sigma‐Aldrich St. Louis, MO, USA), washed twice with PBS/1% BSA, and stained overnight with an anti‐LDL receptor rabbit polyclonal antibody (Abcam, Cambridge, UK) diluted 1:400 in PBS/1% BSA. The cells were washed twice with PBS/1% BSA and then incubated with an Alexa‐Fluor 488‐conjugated goat anti‐rabbit secondary antibody diluted 1:500 in PBS/1% BSA (Thermo Fisher Scientific Waltham, MA, USA). Nuclei were counterstained with Hoechst 3342 (Thermo Fisher Scientific). Confocal microscopy analysis was performed using a Zeiss LSM 900 confocal microscope equipped with ZEN 3.2 Blue Edition software.

### 2.11 Dil‐LDL uptake assay

HepG2 cells were seeded on 24‐well plates by Corning Incorporated (Corning, NY, USA). At 70% confluency, cells were incubated for 24 h before the experiment with MEM supplemented with lipoprotein‐deficient serum from fetal calf, and then treated with 7 μg/mL PCSK9 alone or in combination with 0.1 μM HFn‐Pep2‐8 and 1 μM HFn‐Pep2‐8 in serum‐free medium. After 6 h, the treated cells were washed with DPBS before the addition of fresh serum‐free MEM containing purified human plasma low‐density lipoprotein (LDL) Dil‐labeled, by Alpha Diagnostic International (San Antonio, TX, USA) at a final concentration of 20 μg/mL. After 2 h of incubation, the cells were fixed with 4% paraformaldehyde for 10 min, washed with DPBS, and analyzed by confocal microscopy using a Zeiss LSM 900 confocal microscope equipped with ZEN 3.2 Blue Edition software. Twelve fields from each well were examined, and Dil‐LDL uptake was measured as the MFI.

## 3 RESULTS AND DISCUSSION

**FIGURE 1:**
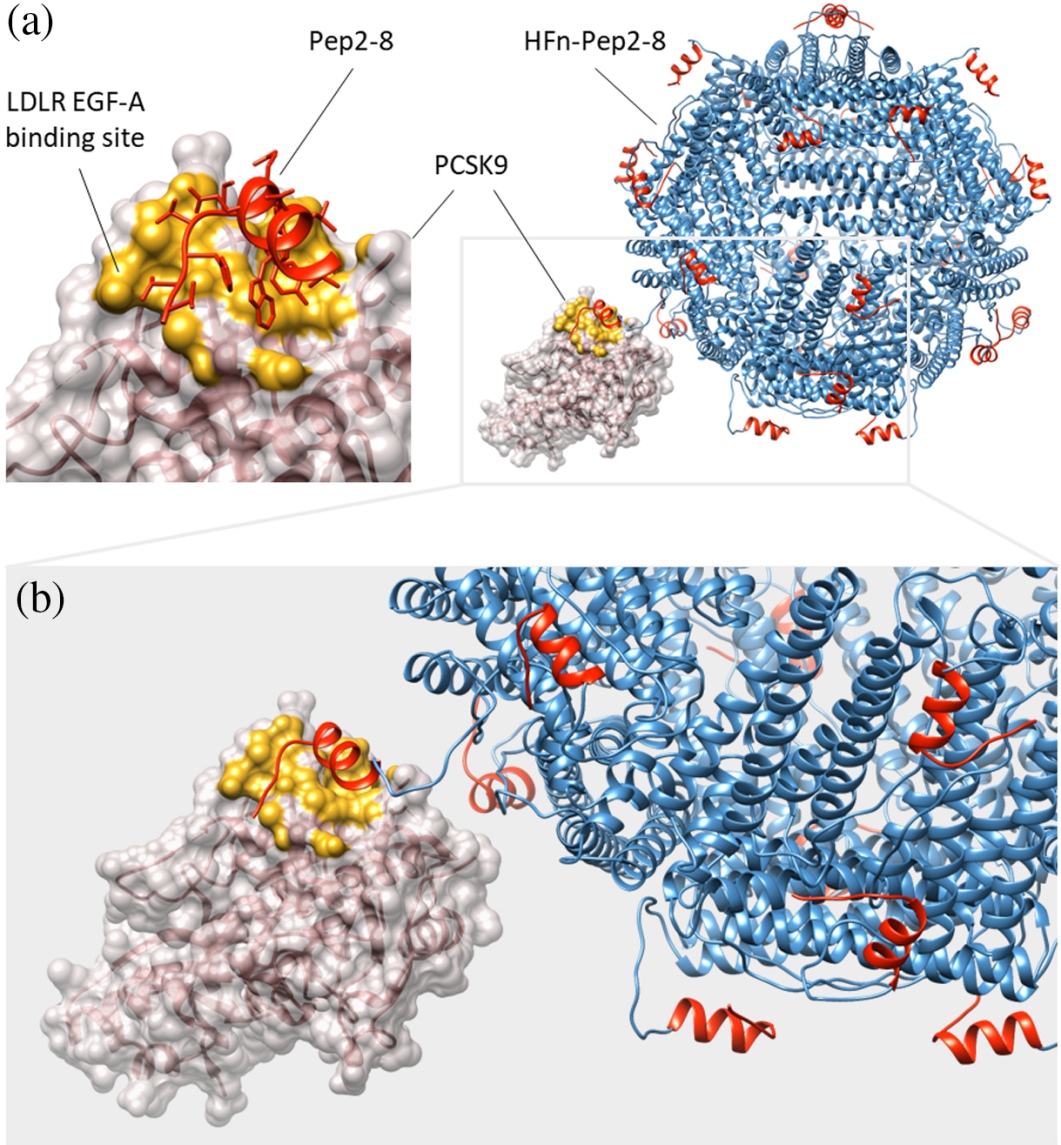
Interactions of Pep2‐8 and HFn‐Pep2‐8 with PCSK9. (a) Pep2‐8 (red), fused to the N‐terminal region of HFn (blue) (PDB entry 2FHA) binds to the PCSK9 catalytic domain in the region involved in LDLR recognition (yellow) (PDB entry 4NMX). (b) Alphafold‐generated 3D model of the interaction of HFn‐Pep2‐8 with PCSK9.

This study aimed to genetically modify a human H ferritin nanoparticle to recognize PCSK9, a protein involved in cholesterol metabolism (Seidah & Prat, 2022), by adding a peptide sequence at the *N*‐terminus of each subunit of the protein (Figure 1). The peptide sequence was selected based on previously reported PCSK9‐targeting peptide sequences obtained through combinatorial library screening of randomized peptide sequences (Zhang et al., 2014). From the pool of screened peptides, we selected Pep2‐8 (TVFTSWEEYLDWV), which forms a ß‐strand‐turn‐helix motif that binds to an exposed, slightly convex region on the PCSK9 catalytic domain (Kwon et al., 2008). This region is a potentially druggable site, suitable for the binding of antibodies and peptides aimed at preventing PCSK9's interaction with the EGF‐A domain of LDLR. (Figure 1).

Biolayer interferometry studies showed that Pep2‐8 had moderate affinity for PCSK9 (*K*_D_ = 0.6610 μM) (Zhang et al., 2014). Despite this, Pep2‐8 was selected for this study due to its shorter length (13 amino acids) and the lack of cysteine residues, ensuring structural stability and reducing oxidation risks within the chimeric system. Pep2‐8 was fused to the *N*‐terminus of the protein using a flexible linker composed of four glycine residues (Figure S1).

### 3.1 HFn‐Pep2‐8 characterization

**FIGURE 2:**
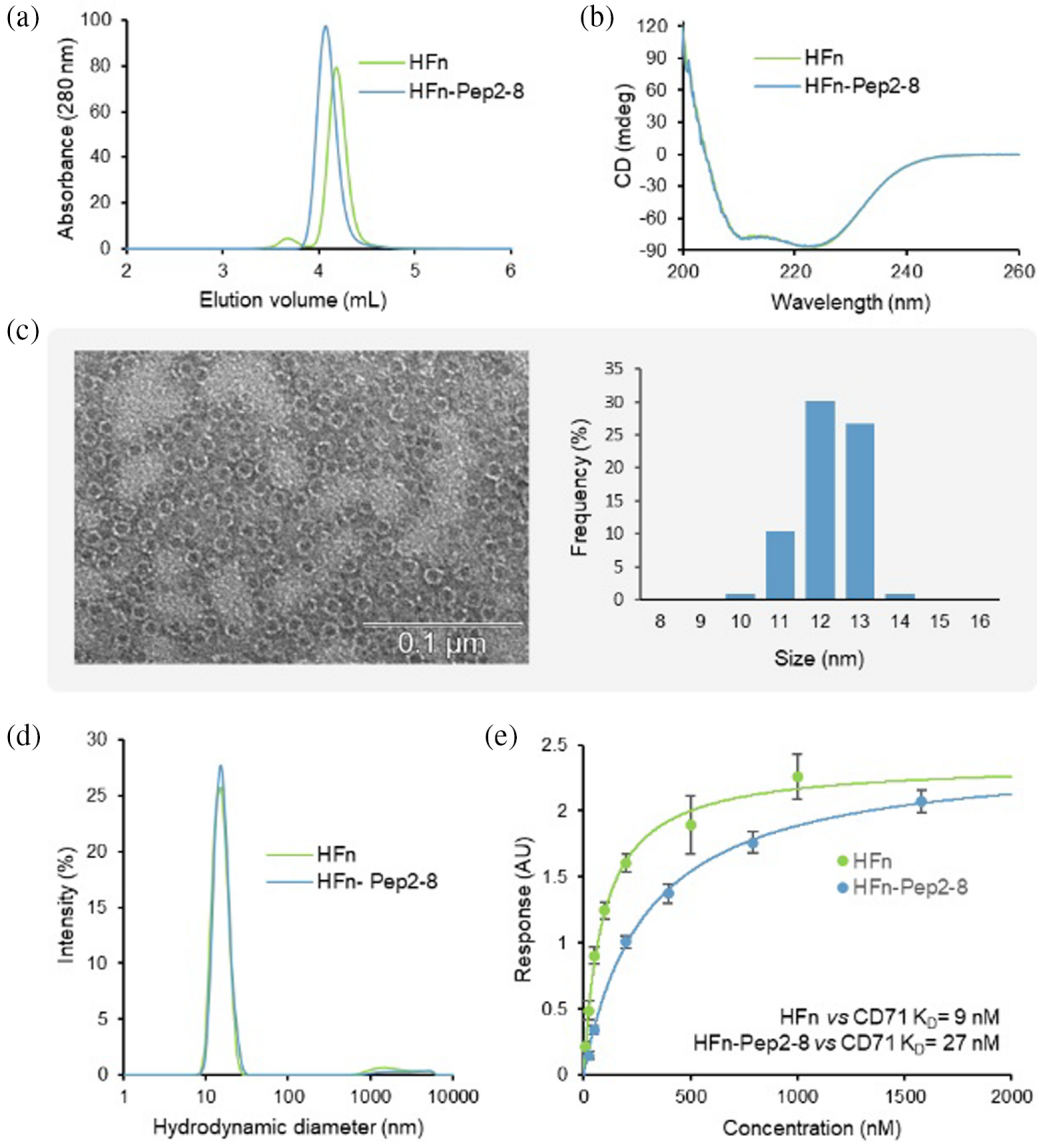
Biochemical characterization of HFn‐Pep2‐8. (a) HP‐SEC chromatogram of HFn‐Pep2‐8 compared to that of HFn. (b) CD spectrum of HFn‐Pep2‐8 compared to that of HFn. (c) Negative TEM staining and diameter distributions of HFn‐Pep2‐8 at a protein dilution of 1.5 μg /mL (scale bar: 0.1 μm). (d) Size distribution of HFn and HFn‐Pep2‐8 measured by DLS analysis. (e) Equilibrium dissociation constants (*K*_D_) of the interactions of HFn and HFn‐Pep2‐8 with the CD71 receptor obtained by BLI experiments. The obtained *K*_D_ values are shown in the graphs for each complex. Binding assays were performed in duplicate.

The synthetic gene encoding for HFn‐Pep2‐8 was optimized for the expression in *E. coli* cells, and the recombinant protein was expressed at a high level (20 mg of purified protein per liter of culture). The purified protein was obtained from the inclusion bodies, as described in Figure S2. HP‐SEC, electrophoresis, CD, DLS, TEM, BLI, and MALDI TOF/TOF analyses were utilized to comprehensively characterize HFn‐Pep2‐8 (Figure 2, Figures S3 and S4).

HP‐SEC analysis (Figure 2a) revealed that the protein was highly pure and properly assembled and eluted as a single peak with a lower retention volume than that of unmodified ferritin due to its higher molecular weight. This finding provides strong evidence of an extra sequence added at each ferritin subunit. MALDI TOF/TOF analysis confirmed the presence of the Pep2‐8 peptide and the linker at the *N*‐terminus, providing information on the identity and location of the modification (Figure S4). Notably, the PCSK9‐recognizing domain did not alter the secondary structure of ferritin, as revealed by CD analysis (double minima at 210 and 222 nm), suggesting that the modification did not cause any significant changes in the alpha helix content of the protein (Figure 2b). TEM analysis revealed a homogenous distribution of the modified ferritin nanoparticles in the sample, with a typical donut shape morphology, characterized by a diameter size distribution ranging from 11 to 13.5 nm (average of 12.12 ± 0.6 nm), which is consistent with previous reports on unmodified ferritins (Lawson et al., 1991) (Figure 2c). These results are in agreement with the DLS data. The size distributions showed narrow peaks at approximately 15.1 ± 0.1 nm and 15.8 ± 0.6 nm for the HFn and HFn‐Pep2‐8 suspensions, respectively (Figure 2d). The slight increase in the hydrodynamic diameter is probably due to the Pep2‐8 peptide being linked to the HFn surface. The hydrodynamic diameters (*Z*‐average values) were 15.1 ± 0.1 nm for HFn and 15.8 ± 0.6 nm for HFn‐Pep2‐8. The polydispersity index values obtained by DLS analysis (0.166 ± 0.008 and 0.179 ± 0.007 for the HFn and HFn‐Pep2‐8 nanoparticles, respectively) highlighted monodisperse suspensions, as also confirmed by multi‐angle DLS (MADLS). This technique, which combines the scattering angle information from Mie theory and the particle size distribution analysis from a DLS measurement, shows a single population for both nanoparticles. The HFn suspensions consisted of 1.15 × 10^+14^ ± 0.08 × 10^+14^ nanoparticles with a hydrodynamic diameter of 14.0 nm, and the HFn‐Pep2‐8 suspension consisted of 7.06 × 10^+13^ ± 0.49 × 10^+13^ nanoparticles with a hydrodynamic diameter of 14.5 ± 0.7 nm.

The values of the zeta potential (−12.9 ± 2.7 mV for HFn and − 6.8 ± 1.5 mV for HFn‐Pep2‐8) indicated a negative surface charge for both nanoparticles. While the net negative charge of HFn‐Pep2‐8 is indeed higher compared to HFn, the difference in zeta potential may stem from variations in the compactness of the counter‐ion layer, differences in charge distribution, surface roughness, and the hydration layer. These factors contribute to distinct electrical potentials at the slipping plane, which separates mobile fluid from fluid that remains attached to the surface.

Overall, these findings suggest that the modified ferritin retains the ability to self‐assemble properly, a critical factor in maintaining stability and preventing aggregation in solution. The accuracy of the assembly was further confirmed by its capacity to recognize the CD71 receptor, as demonstrated through biolayer interferometry (Figure 2e and Figure S5), a powerful tool for real‐time measurement of biomolecular interactions. BLI experiments were conducted using the His‐tagged CD71 receptor immobilized on the biosensor tip, revealing a *K*_D_ value of 9 nM for human ferritin, which is consistent with values reported in other publications (Montemiglio et al., 2019). In contrast, HFn‐Pep2‐8 exhibited a slightly lower affinity, with a *K*_D_ value of 27 nM.

When developing nanoparticles for potential therapeutic applications, a crucial step involves evaluating their storage stability and resilience across a spectrum of experimental conditions. In the specific context of the HFn‐Pep2‐8 construct, the incorporation of the Pep2‐8 sequence at the *N*‐terminus via a flexible linker introduces potential vulnerability to proteolytic cleavage. Consequently, it is essential to investigate the likelihood of alterations occurring over time in both the *N*‐terminal region and the overall quaternary structure. SDS‐PAGE analysis demonstrated that HFn‐Pep2‐8 was not proteolyzed for up to 12 months in phosphate buffer supplemented with sodium chloride, sterile‐filtered, and stored at 4°C (Figure S6). Additionally, UV spectra confirmed that the protein is perfectly soluble within the same timeframe. However, to evaluate its stability within the cell culture environment, HFn‐Pep2‐8 was incubated at 37°C in MEM, a culture medium commonly used to grow the HepG2 liver cell line, an established model for studying cholesterol metabolism. Under these conditions, the protein was stable for up to 8 h.

### 3.2 HFn‐Pep2‐8 interaction with PCSK9

**FIGURE 3:**
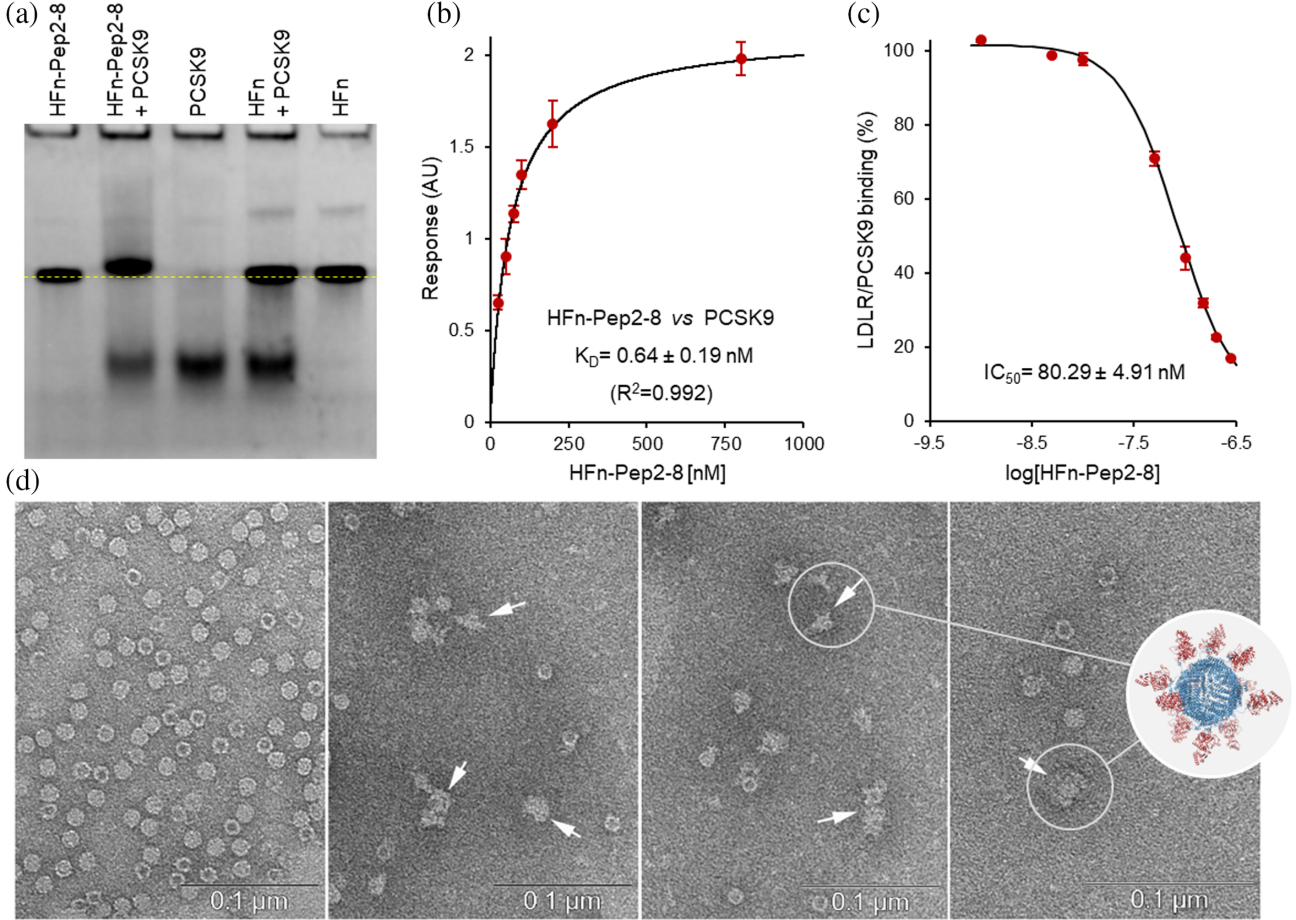
HFn‐Pep2‐8 interaction with PCSK9. (a) Native PAGE of HFn (0.36 μM) and HFn‐Pep2‐8 (0.36 μM) alone or incubated overnight at 4°C with a sevenfold excess of PCSK9 (2.6 μM). PCSK9 was also run as a reference. The yellow dotted line highlights the mobility shift of ferritin nanoparticles. (b) Equilibrium dissociation constants (*K*_D_) of the interactions of HFn‐Pep2‐8 with immobilized PCSK9 obtained by BLI experiments. The *K*_D_ value presented in the graph represents the result of binding assays performed in duplicate. (c) Determination of the in vitro median inhibitory concentration (IC_50_) of HFn‐Pep2‐8 in the assay of PCSK9‐binding to LDLR. The results are presented as the mean ± SD (*n* = 3). (d) Negative TEM staining of HFn‐Pep2‐8 (1.5 μg/mL) alone or preincubated for 1 h at 37°C with a 10‐molar excess of PCSK9 (scale bar: 0.1 μm). The inset shows a 3D model of HFn‐Pep2‐8 (blue) interacting with PCSK9 (red).

The first evidence of the interaction between HFn‐Pep2‐8 and PCSK9 was obtained through high‐performance size exclusion chromatography (Figure S7) and native gel electrophoresis (Figure 3a). A distinct electrophoretic mobility shift is observed when PCSK9 is incubated with either HFn or HFn‐Pep2‐8. In the latter case, the formation of a complex is evidenced by a shift in the ferritin band and a reduction in the intensity of the PCSK9 band. Notably, these changes are not observed when PCSK9 is incubated with HFn, indicating specificity in the interaction.

The strength of HFn‐Pep2‐8–PCSK9 interaction was evaluated through both biolayer interferometry and ELISA measurements. In the BLI analysis (Figure 3b and Figure S8), the His‐tagged PCSK9 protein was immobilized on a biosensor tip, and various concentrations of ferritin were tested.

Our results show that HFn‐Pep2‐8 exhibits a high affinity for the PCSK9 protein, with a dissociation constant (*K*_D_) of 0.64 ± 0.19 nM, approximately 1000‐fold lower than that of the free Pep2‐8 peptide (Zhang et al., 2014).

This strong binding capacity to PCSK9 can be explained through several synergistic effects. First, ferritin acts as a multivalent scaffold, presenting 24 Pep2‐8 peptides in a dense and highly symmetric manner. This multivalency significantly enhances the overall binding strength due to the cumulative effect of multiple simultaneous interactions between the Pep2‐8 peptides and PCSK9. In addition, the increased local concentration of Pep2‐8 peptides around the PCSK9 molecule also plays a crucial role. When Pep2‐8 peptides are linked to ferritin, they are concentrated in close proximity to each other, creating a high local concentration of binding sites. This proximity effect increases the likelihood of the interaction with PCSK9, leading to more frequent and robust binding. Furthermore, in a multivalent system such as HFn‐Pep2‐8, if PCSK9 dissociates from one of the 24 Pep2‐8 peptides displayed on the nanoparticle surface, the remaining peptides can still maintain the overall binding interaction, resulting in a higher apparent affinity and greater stability of the binding interaction.

Notably, while the *K*_D_ value reported in the literature for evolocumab, an FDA‐approved monoclonal antibody used to treat hypercholesterolemia, is significantly lower at 16 pM, alirocumab, another FDA‐approved treatment, has a *K*_D_ value of 0.52 nM, which is very similar to that found for HFn‐Pep2‐8. However, it is important to interpret any comparison between these values with caution, as the K_D_ values for evolocumab and alirocumab were obtained using different experimental setups.

Pep2‐8 peptide acts as a competitive inhibitor as its binding to PCSK9 hinders the interaction of PCSK9 with the EGF‐A domain of LDLR (Figure 1) (Zhang et al., 2014). Thus, to evaluate the ability of HFn‐Pep2‐8 to disrupt the in vitro PCSK9–LDLR interaction, we performed an ELISA test with the LDLR‐AB domain pre‐coated onto plates. As shown in the Figure 3c, HFn‐Pep2‐8 showed a dose‐dependent inhibition with a half‐maximal inhibition (IC_50_) in the low nanomolar range (80.29 ± 4.91 nM).

The BLI and ELISA results were supported by TEM negative stain analysis of HFn‐Pep2‐8 nanoparticles incubated with PCSK9 (Figure 3d). The images clearly show the presence of thickened material partially or completely surrounding ferritin nanoparticles in the treated samples (arrows), clearly indicating that the PCSK9 molecules interact with HFn‐Pep2‐8. The observed heterogeneity is likely due to the intrinsic variability of nanoparticle‐based systems, where multiple binding events can happen either simultaneously or in sequence, resulting in a distribution of bound states. Indeed, Pep2‐8 is connected to HFn via an unstructured glycine linker, which imparts flexibility to the peptide exposed on the surface. This flexibility is advantageous for molecular recognition but also makes the interaction statistically variable, making it challenging to establish the exact stoichiometry. Although ferritin displays 24 binding sites for PCSK9, the number of PCSK9 molecules bound to the nanoparticles will likely be lower than the available binding sites due to the dimensions of PCSK9 and steric hindrance. One of the major challenges in determining the exact stoichiometry is that high concentrations of PCSK9 (>50 μM) are required. Under these conditions, far from the physiological ones, PCSK9 tends to aggregate, limiting this kind of investigation.

### 3.3 In vitro cell experiments

Based on comprehensive biochemical characterization, we conducted tests to assess the impact of HFn‐Pep2‐8 on the HepG2 liver cell line. Given the role of high PCSK9 levels in diminishing LDLR expression on the surface of liver cells (Kwon et al., 2008), our investigation aimed to elucidate the potential of HFn‐Pep2‐8 treatment in modulating membrane receptor levels. PCSK9 is the main therapeutic target of the most innovative biological therapies for the treatment of hypercholesterolemia. The lowering of PCSK9 levels is currently being pursued through the use of monoclonal antibodies (evolocumab and alirocumab) and small interfering RNAs (inclisiran), with significant improvements in the clinical picture, especially for statin‐resistant subjects. The present study fits into this context by utilizing the smallest peptide known to inhibit the activity of PCSK9, thereby increasing its effectiveness by exploiting the multivalent effect of ferritin. Indeed, when Pep2‐8 is fused to each ferritin subunit, the resulting nanoparticle has a *K*_D_ 1000 times lower than the free peptide. This ferritin‐based nanoparticle was then tested on HepG2 liver cells, a model system for anti‐hypercholesterolemic drug screening. First, we determined that HFn‐Pep2‐8 had no toxic effects on HepG2 cells at the concentrations used (Figure S9). Then, we added PCSK9 alone (7 μg/mL) or in combination with different amounts of HFn‐Pep2‐8 (0.1 and 1 μM) to the HepG2 culture medium for 8 h. Confocal microscopy was used to visualize LDLR expression through immunofluorescent labeling.

**FIGURE 4:**
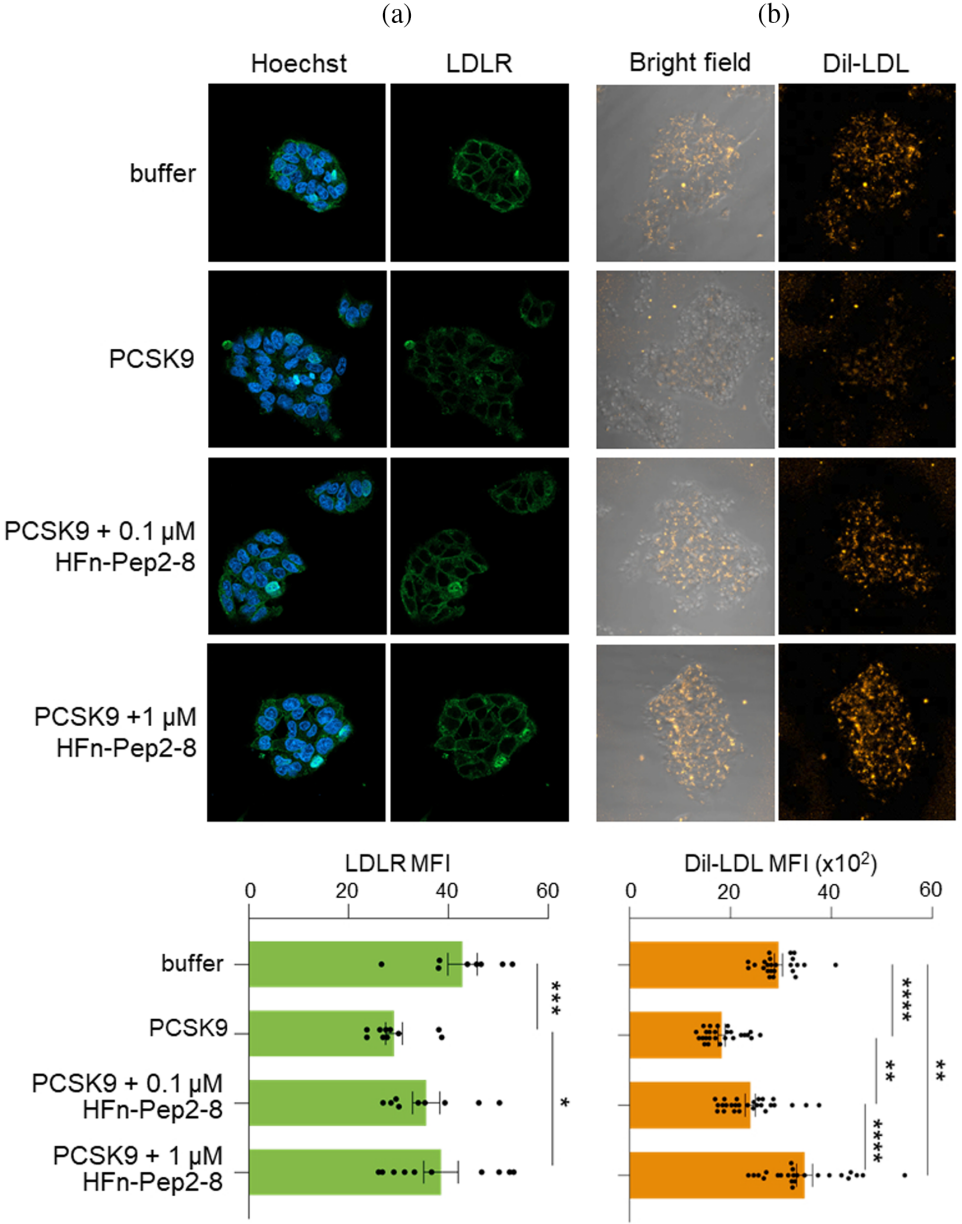
HFn‐Pep2‐8 rescues the loss of LDL receptors at the plasma membrane induced by PCSK9. (a) HepG2 cells were incubated for 8 h with 7 μg/mL PCSK9 alone or in combination with 0.1 μM or 1 μM HFn‐Pep2‐8 and then fixed and stained to detect LDLR at the plasma membrane. LDLR is labeled in green, and nuclei were counterstained with Hoechst (blue). The graph on the bottom shows the average medium fluorescence intensity (MFI) (*n* = 2 ± standard error of the mean, each dot represents the fluorescence quantification from a field). (b) The same cells were treated as in A for 6 h, then washed and incubated for an additional 2 h with LDL labeled with the lipophilic fluorescent dye Dil. Images in bright field are presented to identify the cells, and Dil‐LDL is shown in orange. The graph on the bottom shows the average MFI (*n* = 2 ± standard error of the mean, each dot represents the fluorescence quantification from a field).

HepG2 cells incubated with PCSK9 alone exhibited significantly lower levels of LDLR on the plasma membrane compared to control cells (Figure 4a), consistent with expectations. Specifically, exposure to 7 μg/mL PCSK9 resulted in a reduction of LDLR protein levels by 32.07% compared to the control cells. This finding is in line with previously published reports (Lammi et al., 2019; Lipari et al., 2012). Notably, supplementation of the culture medium with HFn‐Pep2‐8 reversed LDLR expression in a dose‐dependent manner, with a notable restoration of 90% of the receptors on the HepG2 surface observed at the highest concentration of HFn‐Pep2‐8 tested.

To assess HFn‐Pep2‐8's impact on the capacity of HepG2 cells to uptake extracellular LDL, functional experiments were performed utilizing Dil‐labeled LDL. Cells were treated with PCSK9 alone or in combination with varying concentrations of HFn‐Pep2‐8. Consistent with the reduction in LDLR expression, the uptake of labeled LDL molecules was significantly diminished by 38.13% in the presence of PCSK9. However, supplementation with HFn‐Pep2‐8 (Figure 4b) notably enhanced the uptake to 81.12% at 0.1 μM concentration, with complete restoration achieved at 1 μM concentration (+117.46%). These results demonstrate the efficient interaction of HFn‐Pep2‐8 with PCSK9, antagonizing its binding to LDLR and thereby prolonging its half‐life on the liver cell surface, consequently facilitating LDL uptake.

## 4 CONCLUSIONS

In conclusion, in this study, we successfully engineered human H ferritin nanoparticles to incorporate a PCSK9‐targeting peptide, which demonstrated 1000‐fold stronger binding affinity compared to the free Pep2‐8 peptide due to the synergistic effects of multivalency, increased local concentration of peptides, reduced dissociation rates and optimal spatial arrangement. We demonstrate the efficacy of this nanoparticle in mitigating the PCSK9‐mediated impairment of LDL cholesterol cellular uptake by the HepG2 liver cell line.

The multivalent binding effect determined by the 24 Pep2‐8 peptides displayed on the surface of the ferritin shows its potential as a versatile scaffold for therapeutic intervention, particularly in the context of cardiovascular diseases. Furthermore, the broad implications of PCSK9 modulation across various physiological and pathological conditions suggest promising therapeutic applications for this innovative ferritin‐based nanoparticle beyond cardiovascular disorders, extending its potential impact to diverse medical fields such as liver and infectious diseases, cancer, and autoimmune disorders.

Finally, the multifunctional nature of ferritin makes it a versatile platform that can be easily engineered to display different peptides or proteins, offering the possibility of creating multifunctional therapeutics that target multiple pathways.

## AUTHOR CONTRIBUTIONS

**Alessio Incocciati:** Methodology; investigation; writing – review and editing; validation. **Chiara Cappelletti:** Methodology; investigation; writing – review and editing; validation. **Silvia Masciarelli:** Investigation; writing – review and editing; validation; visualization. **Francesca Liccardo:** Investigation; writing – review and editing; validation. **Roberta Piacentini:** Investigation; writing – review and editing; validation. **Alessandra Giorgi:** Writing – review and editing; investigation. **Lucia Bertuccini:** Writing – review and editing; validation; investigation. **Barbara De Berardis:** Writing – review and editing; validation; investigation. **Francesco Fazi:** Funding acquisition; writing – review and editing; resources; project administration. **Alberto Boffi:** Writing – review and editing; funding acquisition; resources; project administration. **Alessandra Bonamore:** Conceptualization; resources; writing – original draft; writing – review and editing; visualization; supervision; project administration; funding acquisition. **Alberto Macone:** Conceptualization; resources; writing – original draft; writing – review and editing; visualization; supervision; project administration; funding acquisition.

## FUNDING INFORMATION

Sapienza – Progetti Dipartimentali code n. RD12318AA8EEA45D “Development of protein‐based nanoparticles for theranostic applications” to Alessandra Bonamore and Alberto Macone. Project Prin 2022 from MUR code n. 2022WC7BL2 to Alberto Boffi. PAN‐HUB project local code n. T4‐AN‐07, “Traiettoria 4 del Piano Sviluppo e Coesione Salute” – FSC 2014–2020 to Alberto Boffi. NextGenerationEU DD. 3175/2021 E DD. 3138/2021 CN_3: National Center for Gene Therapy and Drugs based on RNA Technology – CN 00000041to Francesco Fazi and Alberto Boffi.

## CONFLICT OF INTEREST STATEMENT

The authors declare that they have no competing interests.

## Supporting information


Figures S1‐S9.

## Data Availability

All data related to the manuscript are available in the manuscript and in the supplementary information in the form of graphs and figures.

## References

[pro5111-bib-0001] Bao X , Liang Y , Chang H , Cai T , Feng B , Gordon K , et al. Targeting proprotein convertase subtilisin/kexin type 9 (PCSK9): from bench to bedside. Signal Transduct Target Ther. 2024;9(1):13. 10.1038/s41392-023-01690-3 38185721 PMC10772138

[pro5111-bib-0002] Calisti L , Cardoso Trabuco M , Boffi A , Testi C , Montemiglio LC , des Georges A , et al. Engineered ferritin for lanthanide binding. PLoS One. 2018;13(8):e0201859. 10.1371/journal.pone.0201859 30102720 PMC6089422

[pro5111-bib-0003] Cohen JC , Boerwinkle E , Mosley TH , Hobbs HH . Sequence variations in PCSK9, low LDL, and protection against coronary heart disease. N Engl J Med. 2006;354:1264–1272. 10.1056/NEJMoa054013 16554528

[pro5111-bib-0004] Houser KV , Chen GL , Carter C , Crank MC , Nguyen TA , Burgos Florez MC , et al. Safety and immunogenicity of a ferritin nanoparticle H2 influenza vaccine in healthy adults: a phase 1 trial. Nat Med. 2022;28:383–391. 10.1038/s41591-021-01660-8 35115706 PMC10588819

[pro5111-bib-0005] Huang CW , Chuang CP , Chen YJ , Wang HY , Lin JJ , Huang CY , et al. Integrin α2β1‐targeting ferritin nanocarrier traverses the blood–brain barrier for effective glioma chemotherapy. J Nanbiotechnol. 2021;19:180. 10.1186/s12951-021-00925-1 PMC8201891 34120610

[pro5111-bib-0006] Incocciati A , Kubeš J , Piacentini R , Cappelletti C , Botta S , Bertuccini L , et al. Hydrophobicity‐enhanced ferritin nanoparticles for efficient encapsulation and targeted delivery of hydrophobic drugs to tumor cells. Protein Sci. 2023;32(12):e4819. 10.1002/pro.4819 37883077 PMC10661074

[pro5111-bib-0007] Jiang L , Wang LY , Cheng XS . Novel approaches for the treatment of familial hypercholesterolemia: current status and future challenges. J Atheroscler Thromb. 2018;25(8):665–673. 10.5551/jat.43372 29899171 PMC6099065

[pro5111-bib-0008] Joyce MG , King HAD , Elakhal‐Naouar I , Ahmed A , Peachman KK , Macedo Cincotta C , et al. A SARS‐CoV‐2 ferritin nanoparticle vaccine elicits protective immune responses in nonhuman primates. Sci Transl Med. 2022;14:eabi5735. 10.1126/scitranslmed.abi5735 34914540

[pro5111-bib-0009] Kwon HJ , Lagace TA , McNutt MC , Deisenhofer J . Molecular basis for LDL receptor recognition by PCSK9. PNAS. 2008;105(6):1820–1825. 10.1073/pnas.0712064105 18250299 PMC2538846

[pro5111-bib-0010] Lammi C , Sgrignani J , Arnoldi A , Grazioso G . Biological characterization of computationally designed analogs of peptide TVFTSWEEYLDWV (Pep2‐8) with increased PCSK9 antagonistic activity. Sci Rep. 2019;9:2343. 10.1038/s41598-018-35819-0 30787312 PMC6382862

[pro5111-bib-0011] Lawson DM , Artymiuk PJ , Yewdall SJ , Smith JMA , Livingstone JC , Treffry A , et al. Solving the structure of human H ferritin by genetically engineering intermolecular crystal contacts. Nature. 1991;349:541–544. 10.1038/349541a0 1992356

[pro5111-bib-0012] Lee NK , Cho S , Kim IS . Ferritin – a multifaceted protein scaffold for biotherapeutics. Exp Mol Med. 2022;54:1652–1657. 10.1038/s12276-022-00859-0 36192487 PMC9527718

[pro5111-bib-0013] Li L , Muñoz‐Culla M , Carmona U , Lopez MP , Yang F , Trigueros C , et al. Ferritin‐mediated siRNA delivery and gene silencing in human tumor and primary cells. Biomaterials. 2016;98:143–151. 10.1016/j.biomaterials.2016.05.006 27187278

[pro5111-bib-0014] Libby P , Tokgözoğlu L . Chasing LDL cholesterol to the bottom – PCSK9 in perspective. Nat Cardiovasc Res. 2022;1:554–561. 10.1038/s44161-022-00085-x 39195874

[pro5111-bib-0015] Lipari MT , Li W , Moran P , Kong‐Beltran M , Sai T , Joyce Lai S , et al. Furin‐cleaved proprotein convertase subtilisin/kexin type 9 (PCSK9) is active and modulates low density lipoprotein receptor and serum cholesterol levels. J Biol Chem. 2012;287:43482–43491. 10.1074/jbc.M112.380618 23135270 PMC3527935

[pro5111-bib-0016] Ma Y , Dong Y , Li X , Wang F , Zhang Y . Tumor‐penetrating peptide‐functionalized ferritin enhances antitumor activity of paclitaxel. ACS Appl Bio Mater. 2021;4(3):2654–2663. 10.1021/acsabm.0c01613 35014305

[pro5111-bib-0017] Ma Y , Li R , Dong Y , You C , Huang S , Li X , et al. tLyP‐1 peptide functionalized human H chain ferritin for targeted delivery of paclitaxel. Int J Nanomed. 2021;4(16):789–802. 10.2147/IJN.S289005 PMC7869709 33568906

[pro5111-bib-0018] Macone A , Masciarelli S , Palombarini F , Quaglio D , Boffi A , Cardoso Trabuco M , et al. Ferritin nanovehicle for targeted delivery of cytochrome C to cancer cells. Sci Rep. 2019;9:11749. 10.1038/s41598-019-48037-z 31409839 PMC6692331

[pro5111-bib-0019] Montemiglio LC , Testi C , Ceci P , Falvo E , Pitea M , Savino C , et al. Cryo‐EM structure of the human ferritin–transferrin receptor 1 complex. Nat Commun. 2019;10:1121. 10.1038/s41467-019-09098-w 30850661 PMC6408514

[pro5111-bib-0020] Neerathilingam M , Mysore S , Gandham SHA . Soni‐removal of nucleic acids from inclusion bodies. Biochem Biophys Res Commun. 2014;448(1):45–49. 10.1016/j.bbrc.2014.04.049 24747565

[pro5111-bib-0021] Palombarini F , Masciarelli S , Incocciati A , Liccardo F , Di Fabio E , Iazzetti A , et al. Self‐assembling ferritin‐dendrimer nanoparticles for targeted delivery of nucleic acids to myeloid leukemia cells. J Nanbiotechnol. 2021;19:172. 10.1186/s12951-021-00921-5 PMC8190868 34107976

[pro5111-bib-0022] Seidah NG , Prat A . The multifaceted biology of PCSK9. Endocr Rev. 2022;43(3):558–582. 10.1210/endrev/bnab035 35552680 PMC9113161

[pro5111-bib-0023] Sevieri M , Mazzucchelli S , Barbieri L , Garbujo S , Carelli S , Bonizzi A , et al. Ferritin nanoconjugates guide trastuzumab brain delivery to promote an antitumor response in murine HER2 + breast cancer brain metastasis. Pharmacol Res. 2023;196:106934. 10.1016/j.phrs.2023.106934 37734460

[pro5111-bib-0024] Shapiro MD , Tavori H , Fazio S . PCSK9: from basic science discoveries to clinical trials. Circ Res. 2018;122(10):1420–1438. 10.1161/CIRCRESAHA.118.311227 29748367 PMC5976255

[pro5111-bib-0025] Sitia L , Sevieri M , Bonizzi A , Allevi R , Morasso C , Foschi D , et al. Development of tumor‐targeted indocyanine green‐loaded ferritin nanoparticles for intraoperative detection of cancers. ACS Omega. 2020;5(21):12035–12045. 10.1021/acsomega.0c00244 32548382 PMC7271044

[pro5111-bib-0026] Song N , Zhang J , Zhai J , Hong J , Yuan C , Liang M . Ferritin: a multifunctional nanoplatform for biological detection, imaging diagnosis, and drug delivery. Acc Chem Res. 2021;54(17):3313–3325. 10.1021/acs.accounts.1c00267 34415728

[pro5111-bib-0027] Tetter S , Hilvert D . Enzyme encapsulation by a ferritin cage. Angew Chem. 2017;56(47):14933–14936. 10.1002/anie.201708530 28902449

[pro5111-bib-0028] Tombling BJ , Zhang Y , Huang YH , Craik DJ , Wang CK . The emerging landscape of peptide‐based inhibitors of PCSK9. Atherosclerosis. 2021;330:52–60. 10.1016/j.atherosclerosis.2021.06.903 34246818

[pro5111-bib-0029] Vu MN , Pilkington EH , Lee WS , Tan HX , Davis TP , Truong NP , et al. Engineered ferritin nanoparticle vaccines enable rapid screening of antibody functionalization to boost immune responses. Adv Healthc Mater. 2023;12(17):2202595. 10.1002/adhm.202202595 PMC11469303 36786027

[pro5111-bib-0030] Zhang Y , Eigenbrot C , Zhou L , Shia S , Li W , Quan C , et al. Identification of a small peptide that inhibits PCSK9 protein binding to the low density lipoprotein receptor. J Biol Chem. 2014;289(2):942–955. 10.1074/jbc.M113.514067 24225950 PMC3887217

[pro5111-bib-0031] Zhao Z , Tuakli‐Wosornu Y , Lagace TA , Kinch L , Grishin NV , Horton JD , et al. Molecular characterization of loss‐of‐function mutations in PCSK9 and identification of a compound heterozygote. Am J Hum Genet. 2006;79(3):514–523. 10.1086/507488 16909389 PMC1559532

