# Supplementary material for: Ferritin‐based disruptor nanoparticles: A novel strategy to enhance LDL cholesterol clearance via multivalent inhibition of PCSK9–LDL receptor interaction
Source: Protein Sci. 2024 Aug 16;33(9):e5111. doi: 10.1002/pro.5111 (PMC11328107; doi:10.1002/pro.5111)
Supplement: Supplementary file 1 — Figures S1‐S9. [file PRO-33-e5111-s001.docx]

**SUPPLEMENTARY MATERIALS**

**Ferritin-based disruptor nanoparticles: a novel strategy to enhance LDL cholesterol clearance via multivalent inhibition of PCSK9/LDL receptor interaction.**

Alessio Incocciati*^1^, Chiara Cappelletti*^1^, Silvia Masciarelli^2^, Francesca Liccardo^2^, Roberta Piacentini^1,5^, Alessandra Giorgi^1^, Lucia Bertuccini^3^, Barbara De Berardis^4^, Francesco Fazi^2^, Alberto Boffi^1^, Alessandra Bonamore^#1^, Alberto Macone^1^

^1^Department of Biochemical Sciences "Alessandro Rossi Fanelli", Sapienza University of Rome, Piazzale Aldo Moro 5, 00185 Rome Italy.

^2^Department of Anatomical, Histological, Forensic & Orthopaedic Sciences, Section of Histology and Medical Embryology, Sapienza University of Rome, Via A. Scarpa, 14-16, 00161 Rome, Italy; laboratory affiliated to Istituto Pasteur Italia-Fondazione Cenci Bolognetti.

^3^Core Facilities, Microscopy Area, Instituto Superiore di Sanita, Via Regina Elena 299, 00161 Rome, Italy;

^4^National Center for Innovative Technologies in Public Health, Istituto Superiore di Sanità, 00161 Rome, Italy

^5^Center for Life Nano Science@Sapienza, Istituto Italiano di Tecnologia, V.le Regina Elena 291, Rome 00161, Italy.

**Figure S1. HFn-Pep2-8 sequence**

**Figure S2. HFn-Pep2-8 Purification**

**Figure S3. Comparison between HFn and HFn-Pep2-8**

**Figure S4. MALDI TOF/TOF analysis of HFn-Pep2-8**

**Figure S5.** **Biolayer interferometry sensorograms of HFn/HFn-Pep2-8 interaction with CD71**

**Figure S6. HFn-Pep2-8 stability**

**Figure S7. HFn-Pep2-8 interaction with PCSK9: HP-SEC analysis**

**Figure S8.** **Biolayer interferometry sensorogram** **HFn-Pep2-8 interaction with PCSK9**

**Figure S9. Effect of HFn-Pep2-8 on HepG2 cell viability**

**Figure S1**

**HFn-Pep2-8 sequence**

A sequence encoding for Pep2-8 peptide followed by a four-glycine flexible linker was added to the 5’-terminal of a synthetic gene encoding for HFn ferritin subunit to generate HFn-Pep2-8.Each nanoparticle subunit consists of an N terminal peptide corresponding to Pep2-8 (red) connected by a four-glycine linker (black) to HFn (blue).

**Figure S2**

**HFn-Pep2-8 purification**

The synthetic gene encoding for HFn-Pep2-8 was optimized for the expression in *E. coli* cells and highly purified. The recombinant protein was expressed at a very high level upon induction with 1 mM IPTG for 16h at 22°C (Figure S2, panel A). Unlike human HFn, HFn-Pep2-8 was not soluble after sonication, thus a new purification protocol was developed which allowed its recovery from the inclusion bodies. The results of the purification process are shown in figure S2, panel B. As the quality and composition of the inclusion bodies affect the refolding yield and the further purification of the recombinant protein, we decided to introduce a further sonication step in the presence of 1 M urea. This step was necessary to weaken the interaction with the nucleic acid allowing in the meantime its solubilization at low urea concentration. The protein was precipitated by diluting the sample in the presence of salts, resuspended in the buffer, and finally purified by size exclusion chromatography (Figure S2, panel C). The protein fractions eluted between 130 and 150 mL were pooled and used for all the experiments.

**Figure S3**

**Comparison between HFn and HFn-Pep2-8.**

Compared to human HFn, HFn-Pep2-8 has a higher molecular weight, as confirmed by SDS-PAGE (left panel). In native electrophoresis (right panel), HFn-Pep2-8 shows a higher mobility due to the increase in negative charges brought by the Pep2-8 peptide.

**Figure S4**

**MALDI TOF/TOF analysis of HFn-Pep2-8**

Tandem mass spectrum of 1-27 peptide (underlined in the box). The y-series ions are indicated to highlight the aminoacidic sequence. The results confirmed the presence at the N-terminus of the Pep2-8 peptide protein followed by the 4-glycine linker.

**
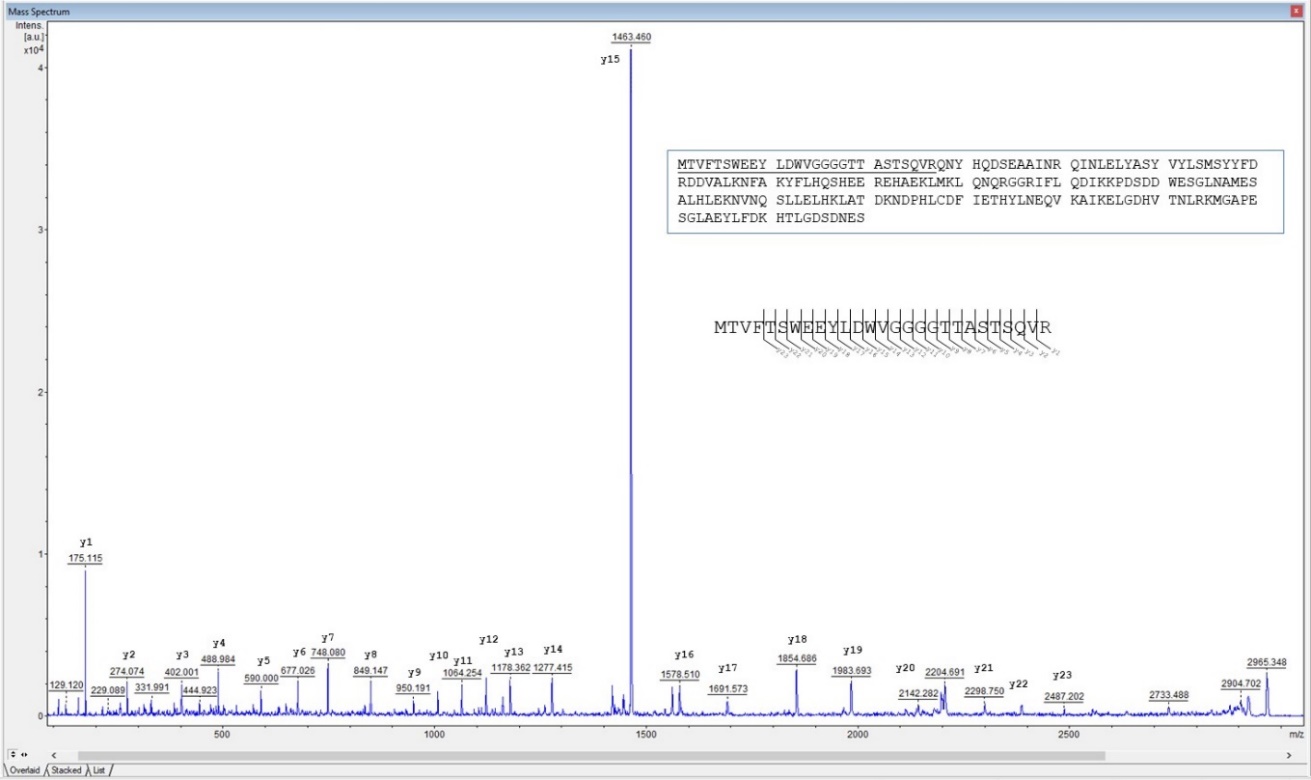
**

**Figure S5**

**Biolayer interferometry sensorograms**  **of HFn/HFn-Pep2-8 interaction with CD71**

The BLI traces show the real time binding of HFn (range 10-1000 nM) or HFn-Pep2-8 (range 25-1580 nM) to His-tagged CD71 immobilized on the biosensor tip. Black lines represent the best fit from Octet software.

**Figure S6**

**HFn-Pep2-8 stability**

(A) SDS-PAGE of purified, sterile filtered HFn-Pep2-8 stored at 4°C. SDS-PAGE was carried out on 12% non-denaturing acrylamide gel (12% Mini-PROTEAN® TGX Stain-Free) using Tris/Gly/SDS as a running buffer up to 12 months.

(B) HP-SEC analysis of HFn-Pep2-8 in MEM medium at 37°C up to 8h. Blue chromatogram: HFn-Pep2-8 in phosphate buffer as a reference; red chromatogram: MEM medium; green chromatogram: HFn-Pep2-8 in MEM after 8 hours incubation at 37°C. The inset graph illustrates the relative abundance of HFn-Pep2-8 in MEM over time, allowing for a quantitative assessment of its stability.

**Figure S7**

**HFn-Pep2-8 interaction with PCSK9: HP-SEC analysis**

To evaluate the interaction between HFn-Pep2-8 and PCSK9, the ferritin nanoparticle was incubated with a 7-fold excess of PCSK9 overnight at 4°C and analyzed by High-Performance Size-Exclusion Chromatography (HP-SEC). HP-SEC measurements were performed on HFn-Pep2-8 and PCSK9 alone and after incubation. Dotted lines on the chromatogram indicate the elution volume of each species present. By reducing the running flow to 0.35 mL/min, it is evident that HFn-Pep2-8 incubated with PCSK9 exhibits a significantly lower elution volume with respect to free HFn-Pep2-8 (9.4695 ± 0.0013 mL *vs* 9.6935 ± 0.0045 mL), confirming the formation of the complex.


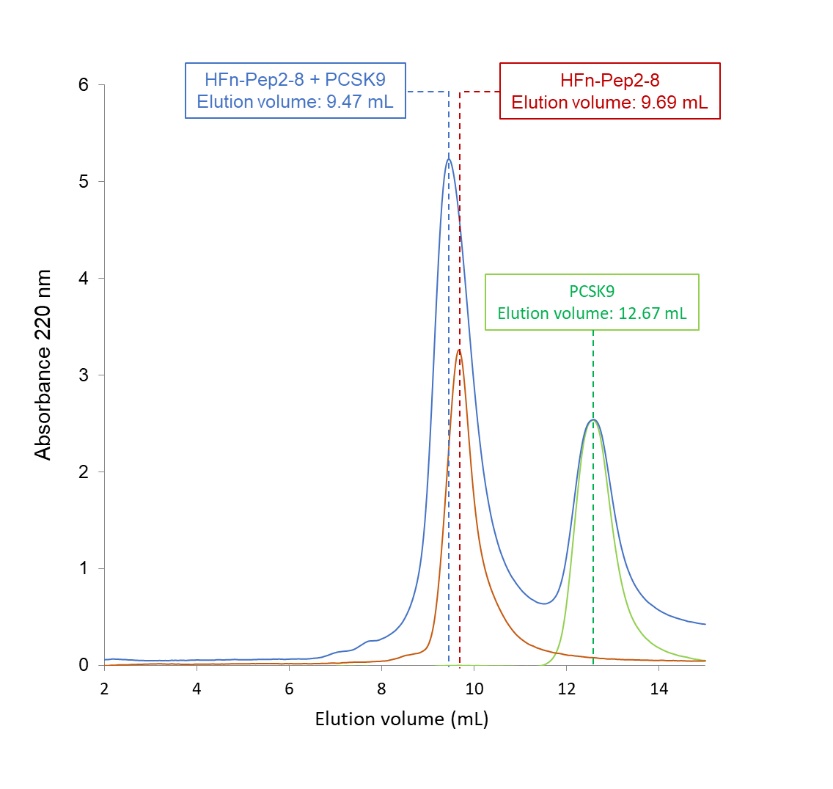


**Figure S8**

**Biolayer interferometry sensorogram** **HFn-Pep2-8 interaction with PCSK9**

The BLI traces show the real time binding of HFn-Pep2-8 (range 10-1000 nM) to His-tagged PCSK9 immobilized on the biosensor tip. Black lines represent the best fit from Octet software.

**Figure S9.**

**HFn-Pep2-8 does not affect HepG2 cell viability.**

HepG2 cells were incubated for 8 h with 0.1 µM or 1 µM HFn-Pep2-8 then detached and stained with propidium iodide (PI), which can enter only damaged/dead cells. The graph reports the average percentage of PI positive cells (n=3 ± SEM).

nil

% PI^+^ cells

0.1µM HFn-Pep2-8

30

20

10

0

1µM HFn-Pep2-8
